# Pharmacokinetics of extrafine beclometasone dipropionate/formoterol fumarate/glycopyrronium bromide in adolescent and adult patients with asthma

**DOI:** 10.1002/prp2.980

**Published:** 2022-06-22

**Authors:** Piotr Kuna, Joanna Jerzynska, Matteo Martini, Andrea Vele, Irene Barneschi, Fabrizia Mariotti, George Georges, Giorgia Ciurlia

**Affiliations:** ^1^ Department of Internal Medicine, Asthma and Allergy Norbert Barlicki Memorial University Hospital No. 1, Medical University of Łódź Łódź Poland; ^2^ Department of Allergology and Child Internal Diseases Medical University of Łódź Copernicus Memorial Hospital, Pediatric Centre Dr. J. Korczak Łódź Poland; ^3^ Global Clinical Development Chiesi Farmaceutici SpA Parma Italy

**Keywords:** adolescents, asthma, pharmacodynamics, pharmacokinetics, systemic exposure, triple therapy

## Abstract

The single‐inhaler extrafine formulation triple combination beclometasone dipropionate (BDP), formoterol fumarate (FF) plus glycopyrronium bromide (GB) is available for asthma management in adults. Its use in adolescents has not yet been evaluated. This study investigated the pharmacokinetic profile of BDP/FF/GB in adults and adolescents, with the aim of ruling out higher plasma exposure in adolescents compared to adults. In this open‐label, non‐randomized study, patients with asthma aged 12–17 (adolescents) and 18–64 years (adults) self‐administered a single dose of BDP/FF/GB 400/24/50 μg via pressurized metered‐dose inhaler (pMDI). The primary objective was to rule out higher systemic exposure to beclometasone 17‐monopropionate (B17MP; active metabolite of BDP), formoterol, and GB in terms of the area under the plasma concentration‐time curve from 0 to the last quantifiable concentration (AUC_0–*t*_) in adolescents versus adults. A total of 40 adolescents and 40 adults entered the study (mean age of 14.8 and 43.6 years, respectively). The primary objective (AUC_0–*t*_) was met, with the upper 90% confidence interval of the geometric mean ratio between adolescents and adults <125% for B17MP (point estimate 79.28 [90% CI 71.19; 88.29]), formoterol (88.68 [77.71; 101.20]) and GB (85.49 [72.96; 100.16]). All secondary pharmacokinetic endpoints supported the primary, with pharmacodynamic (safety) and tolerability results similar in the two populations. In conclusion, systemic exposure to extrafine BDP/FF/GB pMDI in adolescents was not higher than that in adults. Furthermore, there were no safety or tolerability signals to warrant a reduction in the dose of BDP/FF/GB for adolescents with asthma.

## Graphical abstract

Systemic exposure to beclometasone 17‐monopropionate (B17MP; active metabolite of beclometasone dipropionate), formoterol, and glycopyrronium bromide was not higher in adolescents than in adults. All secondary pharmacokinetic endpoints supported the primary, with pharmacodynamic (safety) and tolerability results similar in the two populations. There are, therefore, no signals to warrant a reduction in the dose of extrafine beclometasone dipropionate/formoterol fumarate/glycopyrronium bromide for adolescents with asthma.

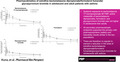

**Abbreviations**
AEs: adverse events
AUC_0–2h_
: area under the plasma concentration‐time curve from 0 to 2 h post–dose
AUC_0–30min_
: area under the plasma concentration‐time curve from 0 to 30 min post–dose
AUC_0–∞_
: area under the plasma concentration‐time curve extrapolated to infinity
AUC_0–*t*_
: area under the plasma concentration‐time curve from 0 to the last quantifiable concentration
B17MP: beclometasone 17‐monopropionate
BDP: beclometasone dipropionate
BMI: body mass index
*C*_max_
: maximum plasma concentration
*C*_min_
: minimum plasma concentration
ECG: electrocardiogram
FEV_1_
: forced expiratory volume in 1 sec
FF: formoterol fumarate
GB: glycopyrronium bromide
HFA: hydrofluoroalkane
HR: heart rate
ICS: inhaled corticosteroid
LABA: long‐acting beta_2_‐agonist
LAMA: long‐acting muscarinic antagonist
PK: pharmacokinetic
pMDI: pressurized metered‐dose inhaler
QTcF: QT interval with Fridericia's correction
*t*_½_
: plasma elimination half‐life
*t*_max_
: time to maximum plasma concentration
*t*_min_
: time to minimum plasma concentration

## 1 INTRODUCTION

For adults or adolescents with asthma that is not controlled by an inhaled corticosteroid/long‐acting beta_2_‐agonist (ICS/LABA) combination, one step‐up maintenance treatment option is the addition of a long‐acting muscarinic antagonist (LAMA) to therapy.^1^ For adults, this can be either using two separate inhalers or single‐inhaler triple therapy. By delivering all three molecules simultaneously, single‐inhaler combinations are easier for patients than using multiple inhalers (often with different instructions for use) and, therefore, may contribute to improved adherence.

One single‐inhaler triple therapy is the extrafine formulation (i.e., with mass median aerodynamic diameter <2 μm) combination of beclometasone dipropionate (BDP), formoterol fumarate (FF), and glycopyrronium bromide (GB). In two 52‐week randomized clinical trials in adults with asthma that was uncontrolled by medium‐ or high‐dose ICS plus LABA, extrafine BDP/FF/GB improved lung function and reduced the rate of moderate‐and‐severe asthma exacerbations compared with extrafine BDP/FF.^2^ In addition, the pharmacokinetic (PK) profile of BDP/FF/GB has previously been investigated in adult healthy volunteers, with the dose‐adjusted PK profile similar for therapeutic and supra‐therapeutic doses, and no evidence of any impact of ethnicity on PK or safety parameters.^3^, ^4^

The efficacy and safety of BDP/FF/GB in adolescents (i.e., aged 12–17 years) have not yet been investigated, and use in this age group is not currently licensed. However, given treatment guidelines do not make a distinction between adults and adolescents in terms of maintenance therapy options,^1^ and no single‐inhaler triple therapy is currently approved for asthma in this age group, the development of BDP/FF/GB for adolescents can help fulfill an unmet need. The current study compared the PK profile of BDP/FF/GB in adults and adolescents and sought to demonstrate that systemic exposure was no higher in adolescents than adults, with a similar safety profile.

## 2 MATERIALS AND METHODS

This was an open‐label, non‐randomized study. Following an initial screening visit, patients attended the study center on Day 1, when they self‐administered the study drug, and on Day 2 when they underwent further post‐dose assessments, with a follow‐up visit 14–16 days after the study drug administration. The study drug consisted of a single dose of BDP/FF/GB, as four inhalations of 100/6/12.5 μg/actuation in 30‐s intervals (total dose of 400/24/50 μg) via hydrofluoroalkane (1,1,1,2‐tetrafluoroethane; HFA‐134a) pressurized metered‐dose inhaler (pMDI). Patients were trained on the correct use of the pMDI both at the screening visit and pre‐dose on Day 1, with the use of a spacer not permitted.

The study recruited male and female non‐ or ex‐smokers who were aged 12–17 years (adolescents) and 18–64 years (adults) who had been diagnosed with asthma, and had a disease that was controlled on medium‐dose ICS (alone or in fixed combination with a LABA), and pre‐bronchodilator forced expiratory volume in 1 s (FEV_1_) >70% predicted. Full inclusion and exclusion criteria are listed in the supplement. All patients (or their parents or legal guardians) provided written informed consent prior to any study‐related procedure. Patients were permitted short‐acting β_2_‐agonists as rescue medication, but not in the 6 h prior to study visits, and ICS (alone or in combination with LABAs) although not in the 2 days before study visits. Use was not permitted in the 3 months prior to screening or during follow‐up of enzyme‐inducing or ‐inhibiting drugs, biologic drugs, or any drug known to have a well‐defined potential for hepatotoxicity. Patients were to avoid smoking or nicotine intake for the entire study period, strenuous activities or food containing poppy seeds from 24‐h pre‐dose until the end of Day 2, and alcohol and xanthine‐containing food or beverages from 48 h pre‐dose until the end of Day 2. There was to be no food intake from 10 h pre‐dose to 2 h post‐dose when a standardized meal was served, and no fluid intake from 1 h pre‐dose to 1 h post‐dose, after which patients were required to drink at least 240 ml of water every 2 h for the following 6 h.

During the treatment period, blood samples were collected pre‐dose and 5, 15, and 30 min, and 1, 2, 4, 8, and 10 h post‐dose for PK assessments, and pre‐dose, and 20 min, and 1, 2, 4 h post‐dose for pharmacodynamic assessments. To determine the PK of BDP and its active metabolite beclometasone 17‐monopropionate (B17MP), blood samples were collected into potassium ethylenediaminetetraacetic acid tubes, then immediately chilled in an ice bath and centrifuged at 4°C, 2500 g for 15 min, with the resulting plasma stored below −20°C until analyzed with a validated liquid chromatography‐tandem mass spectrometry (LC/MS–MS) method, with limits of quantification (LOQ) of 10 pg/ml for BDP and 20 pg/ml for B17MP. For the formoterol and GB evaluations, samples were collected in lithium heparin tubes, and centrifuged at 4°C, 2500 g for 15 min, with the resulting plasma stored at or below −65°C, and analyzed using validated LC/MS–MS methods, with LOQs of 1 pg/ml for both. For potassium and glucose levels, samples were collected into standard serum separating blood collection tubes, allowed to clot at room temperature, then centrifuged at 4°C, 2500 g, for 15 min, with the serum stored below −20°C and then analyzed with photometry for glucose and potentiometry for potassium (both VITROS, Ortho Clinical Diagnostics). In addition, a 12‐lead Holter electrocardiogram (ECG) was used to collect 24 h heart rate and ECG data, with blood pressure data collected up to 24 h post‐dose.

The study was approved by the Bioethics Committee at the Medical University of Łódź, Poland, and was performed in accordance with the principles of the Declaration of Helsinki, and the International Conference on Harmonization notes for guidance on Good Clinical Practice (ICH/CPMP/135/95). The study was registered in the EU Clinical Trials Register (EudraCT number 2019–002238‐35).

### 2.1 Outcomes

Total bioavailability (expressed as the area under the plasma concentration–time curve [AUC] from time 0 to the last quantifiable concentration [AUC_0–*t*_]) reflects pulmonary plus gastrointestinal absorption and is indicative of safety. The primary objective was to rule out a higher systemic exposure to B17MP, formoterol, and GB in terms of AUC_0–*t*_ in adolescent versus adult patients with asthma.

The secondary objectives were: to compare systemic exposure in adolescent versus adult patients in terms of maximum plasma concentration (*C*_max_), time to maximum plasma concentration (*t*_max_), AUC from 0 to 30 min post–dose, and extrapolated to infinity (AUC_0–30min_ and AUC_0–∞_), and plasma elimination half‐life (*t*_½_) for B17MP, formoterol and GB, and AUC_0–*t*_, *C*_max_ and *t*_max_ for BDP; and to evaluate the systemic effects of BDP, B17MP, formoterol, and GB in adolescents with asthma, in terms of heart rate (average 0–4 h, 0–12 h, and 0–24 h), levels of circulating potassium (AUC from 0 to 2 h post–dose [AUC_0–2h_], AUC_0–*t*_, minimum plasma concentration [*C*_min_], and time to minimum plasma concentration [*t*_min_]) and glucose (AUC_0–2h_, AUC_0–*t*_, *C*_max_, *t*_max_), and general safety and tolerability (adverse events [AEs], ECG results [including QT interval with Fridericia's correction, QTcF], and blood pressure).

### 2.2 Sample size and statistical methods

The sample size calculation was based on GB AUC_0–*t*_, the primary PK parameter with the expected highest associated variability. With 74 evaluable patients (37 adults and 37 adolescents), the study would have 80% power to rule out a ratio greater than 125% between adolescents and adults in total systemic exposure, based on a 90% two‐sided confidence interval (CI), assuming an actual ratio of 100% and standard deviation of 0.38 on the log‐scale, with an alpha level of 0.05. Considering a non‐evaluable rate of approximately 7%, a total of 80 patients (40 adults and 40 adolescents) were to be enrolled. The sample size was calculated using PASS version 15.0.5.

AUC_0–*t*_ values were log‐transformed and analyzed using a linear model including patient group (adult or adolescent) as a fixed effect. The ratios of adjusted geometric means between patient groups (adolescent vs. adult) were calculated, and total systemic exposure was considered comparable if the upper limit of the 90% two‐sided CIs of the ratios (adolescent vs. adult) were ≤125%. This cut‐point was based on the upper bound of the 90% CIs when demonstrating bioequivalence (as specified in the European Medicines Agency's *Guideline on the investigation of bioequivalence* ^5^), with the study design (including this cut‐point) agreed with regulatory agencies. The AUC_0–30min_, AUC_0–∞_, *C*_max_, and *t*_½_ values were analyzed using the same model as the primary endpoint; *t*_max_ was analyzed using the Hodges‐Lehmann nonparametric estimate of location shift between patient groups. The PK analyses were performed using WinNonlin Phoenix 6.2 or later (Pharsight Corporation), with *C*_max_ and *t*_max_ obtained directly from the experimental data without interpolation, AUC_0–*t*_ and AUC_0–30min_ computed using the linear trapezoidal rule, and AUC_0–∞_ calculated as the sum of AUC_0–*t*_ and a residual extrapolated to infinite time.

The safety population comprised all enrolled patients who received at least one dose of study treatment; the PK and pharmacodynamic populations comprised all patients from the safety population excluding those without any valid PK or pharmacodynamic measurement, respectively, or with major protocol deviations significantly affecting PK or pharmacodynamic values, respectively.

## 3 RESULTS

### 3.1 Participants

This study was conducted in two clinical centers, both in Poland, between 27 February 2020 and 4 February 2021. A total of 41 adolescents and 41 adults were screened, with 40 of each entering the study, all of whom completed follow‐up. The baseline demographics and disease characteristics of the two age groups are shown in Table 1. Adolescents were more likely to be male, whereas adults were more likely to be female; mean FEV_1_ values were higher in adolescents.

**TABLE 1:** Baseline demographics and disease characteristics of the recruited patients

Parameter	Adolescents (*N* = 40)	Adults (*N* = 40)
Age (years), mean (SD)	14.8 (1.6)	43.6 (14.7)
Sex, male, *n* (%)	25 (62.5)	14 (35.0)
Race, Caucasian, *n* (%)	40 (100)	40 (100)
BMI (kg/m^2^), mean (SD)	22.29 (3.73)	25.25 (3.03)
Non‐smoker, *n* (%)	40 (100)	40 (100)
Time since first asthma diagnosis (years), mean (SD)	7.64 (3.30)	11.54 (7.79)
FEV_1_ (L), mean (SD)	3.51 (0.77)	2.64 (0.81)
FEV_1_% of predicted normal value, mean (SD)	101.4 (14.4)	83.5 (10.4)

Abbreviations: BMI, body mass index; FEV_1_, forced expiratory volume in 1 sec.

### 3.2 Outcomes

#### 3.2.1 Pharmacokinetics

**FIGURE 1:**
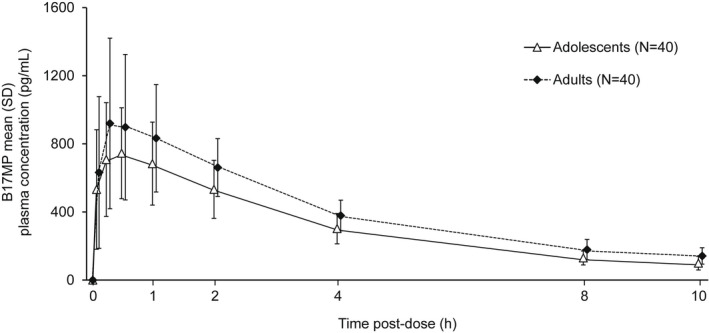
Mean B17MP plasma concentration versus time profiles. B17MP, beclometasone 17‐monopropionate.

**FIGURE 2:**
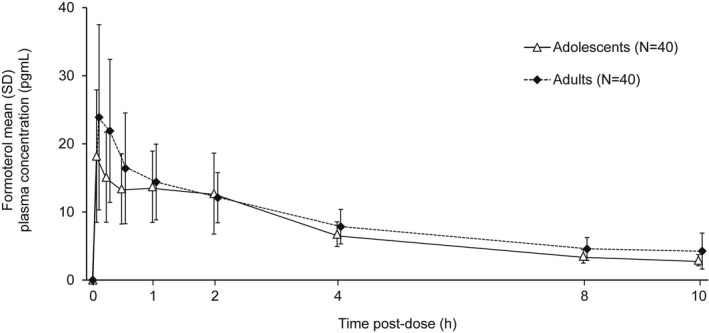
Mean formoterol plasma concentration versus time profiles.

**FIGURE 3:**
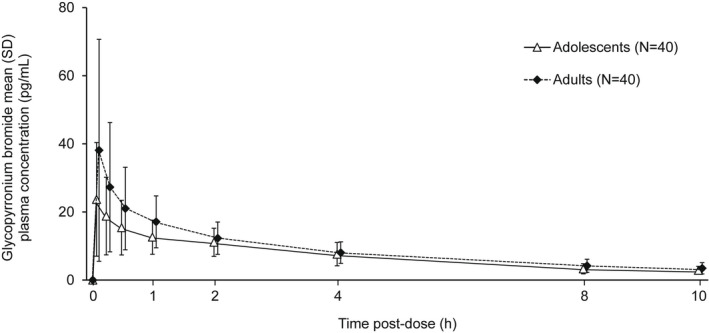
Mean glycopyrronium bromide plasma concentration versus time profiles.

**FIGURE 4:**
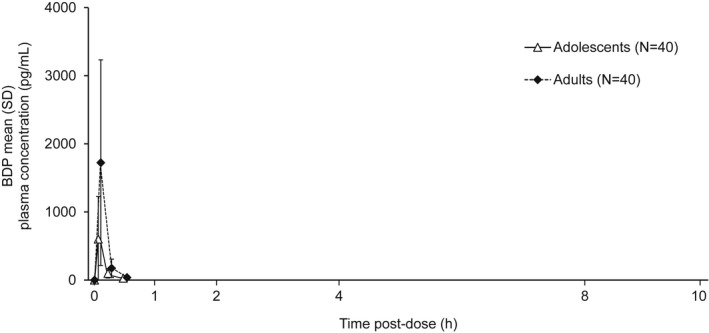
Mean BDP plasma concentration versus time profiles. BDP, beclometasone dipropionate.

Plasma concentration versus time profiles for B17MP, formoterol, GB, and BDP are shown in Figures 1, 2, 3, 4. Overall, the concentration of all four molecules was lower in adolescents than adults, especially at earlier time points. Mean B17MP, formoterol, and GB levels were quantifiable at 10 h post‐dose; BDP was quantifiable only until 30 min post‐dose. The primary objective (AUC_0–*t*_) was met, with the upper 90% CI for the geometric mean ratio between adolescents and adults <125% for B17MP, formoterol, and GB, indicating that exposure was no higher in adolescents than in adults (Table 2). All secondary PK endpoints were consistent with the primary, with systemic exposure again no higher in adolescents than adults, and no difference between the populations for *t*_max_.

**TABLE 2:** Plasma PK parameters, and comparison between the two groups

Parameter	Adolescent (*N* = 40)	Adult (*N* = 40)	Adolescent versus adult adjusted point estimate (90% CI)
B17MP			
*C*_max_ (pg/ml)	831 (328)	1046 (455)^a^	81.07 (68.58; 95.84)
*t*_max_ (h)	0.50 (0.08; 2.00)	0.50 (0.08; 2.02)^a^	0.00 (−0.02; 0.23)
AUC_0–30min_ (h·pg/ml)	307 (134)	381 (205)^a^	86.74 (69.88; 107.67)
AUC_0–*t*_ (h·pg/ml)	3204 (909)	4027 (1022)^a^	79.28 (71.19; 88.29)
AUC_0–∞_ (h·pg/ml)	3660 (1058)^b^	4764 (1282)^c^	76.65 (67.40; 87.16)
*t*_½_ (h)	3.47 (0.633)^d^	4.00 (1.11)^a^	88.47 (81.22; 96.36)
Formoterol			
*C*_max_ (pg/ml)	20.9 (8.27)^b^	27.2 (12.0)^e^	78.83 (65.76; 94.49)
*t*_max_ (h)	0.08 (0.08; 4.00)^b^	0.08 (0.08; 2.02)^e^	0.00 (0.00; 0.00)
AUC_0–30min_ (h·pg/ml)	7.12 (2.95)^b^	9.75 (4.59)^e^	77.87 (62.38; 97.20)
AUC_0–*t*_ (h·pg/ml)	73.8 (21.8)^f^	84.6 (26.6)^e^	88.68 (77.71; 101.20)
AUC_0–∞_ (h·pg/ml)	93.0 (26.5)^g^	92.8 (36.5)^h^	103.95 (82.52; 130.94)
*t*_½_ (h)	4.46 (1.20)^i^	5.25 (1.43)^k^	85.27 (77.04; 94.38)
Glycopyrronium bromide			
*C*_max_ (pg/ml)	25.6 (15.2)	39.5 (31.7)^a^	76.00 (59.92; 96.39)
*t*_max_ (h)	0.08 (0.08; 2.00)	0.08 (0.08; 2.02)^a^	0.00 (0.00; 0.00)
AUC_0–30min_ (h·pg/ml)	8.81 (5.21)	13.3 (9.60)^a^	74.25 (58.44; 94.35)
AUC_0–*t*_ (h·pg/ml)	74.5 (27.5)	90.1 (39.1)^a^	85.49 (72.96; 100.16)
AUC_0–∞_ (h·pg/ml)	91.7 (31.4)^l^	102 (47.7)^m^	93.15 (77.88; 111.42)
*t*_½_ (h)	4.20 (1.66)^a^	4.75 (1.91)^e^	90.04 (79.31; 102.23)
Beclometasone dipropionate			
*C*_max_ (pg/ml)	607 (623)	1724 (1508)	31.56 (22.32; 44.63)
*t*_max_ (h)	0.08 (0.08; 0.25)	0.08 (0.08; 0.27)	0.00 (0.00; 0.00)
AUC_0–*t*_ (h·pg/ml)	111 (118)	282 (235)	35.35 (24.42; 51.17)

*Note*: Adolescent and adult data are arithmetic mean (SD), except *t*_max_, which is median (range). The adolescent versus adult point estimate data are ratios of adjusted geometric means of log‐transformed parameters from the linear model except *t*_max_, which is the Hodges‐Lehmann non‐parametric estimate of location shifts based on untransformed data. *n* = ^a^38, ^b^36, ^c^24, ^d^39, ^e^37, ^f^34, ^g^17, ^h^9, ^i^32, ^k^33, ^l^27, ^m^20. B17MP, beclometasone 17‐monopropionate; *C*_max_, maximum plasma concentration; *t*_max_, time to maximum plasma concentration; AUC, area under the curve from 0 to 30 min (0–30 min), 0 to the last quantifiable concentration (0–*t*), and extrapolated to infinity (0–∞); *t*_½_ plasma elimination half‐life.

#### 3.2.2 Pharmacodynamics

Overall, the pharmacodynamic results were similar in the two populations and provided reassurance about the safety of BDP/FF/GB (Table 3). There were trends to lower mean potassium levels and higher mean glucose levels in adults. Three adults (and no adolescents) had potassium serum concentrations below the normal limit (<3.5 mmol/L), two of whom also had pre‐dose values lower than the normal limit. Six adults had glucose values higher than the normal limit (>5.9 mmol/L) at predose and all post‐dose time points, and two adolescents and 11 adults had glucose values higher than the normal limit at one or more time points between 20 min and 2 h post‐dose. In addition, likely due to the administration of the standardized meal, 28 adolescents and 18 adults had glucose values higher than the normal limit at 4 h post‐dose only. There were no relevant differences between the two populations in heart rate parameters, and changes from baseline were small.

**TABLE 3:** Pharmacodynamic parameters

Parameter	Adolescent (*N* = 38)	Adult (*N* = 37)
Serum potassium		
*C*_min_ (mmol/L)	4.17 (0.370)^a^	3.91 (0.306)^b^
AUC_0–2h_ (h·mmol/L)	8.83 (0.584)^c^	8.34 (0.629)^b^
AUC_0–*t*_ (h·mmol/L)	17.6 (1.14)^d^	16.6 (1.28)^b^
*t*_min_ (h)	2.00 (0.00; 4.00)^e^	2.02 (0.00; 4.05)^b^
Serum glucose		
*C*_max_ (mmol/L)	7.09 (1.49)	7.78 (1.60)
AUC_0–2h_ (h·mmol/L)	10.4 (0.923)	11.3 (1.20)
AUC_0–*t*_ (h·mmol/L)	22.7 (2.33)	25.0 (3.11)
*t*_max_ (h)	4.00 (0.00; 4.08)	4.02 (2.02; 4.05)
Heart rate (bpm)		
HR_0–4h_	81.6 (12.0)	82.8 (11.9)
Change from baseline	4.9 (6.2)	3.7 (6.2)
HR_0–12h_	85.4 (11.6)	84.8 (11.0)
Change from baseline	8.1 (6.2)	6.3 (7.6)
HR_0–24h_	79.9 (9.8)	77.3 (8.5)
Change from baseline	2.8 (6.8)	−1.0 (7.1)

*Note*: Data are arithmetic mean (SD), except *t*_min_ and *t*_max_, which are median (range). *n* = ^a^35, ^b^36, ^c^29, ^d^25, ^e^35. *C*_min_, minimum serum concentration; AUC, area under the curve from 0 to 2 h (0–2 h), and 0 to the last quantifiable concentration (0–*t*); *t*_min_, time to minimum serum concentration; *C*_max_, maximum serum concentration; *t*_max_, time to maximum serum concentration; HR, heart rate.

### 3.3 Safety

Three adults (and no adolescents) had AEs: non‐cardiac chest pain in one patient, anxiety in one patient, and dysgeusia and cough in one patient. These were all considered related to treatment, but were mild in intensity, non‐serious, and resolved with no treatment required before discharge on Day 1. There were no relevant differences between the two age groups in terms of ECG results, and no patients had QTcF interval values >450 ms (males) or >470 ms (females) or changes from baseline >60 ms. Mean changes in blood pressure were generally small, with no relevant differences between age groups. None of the individual ECG or blood pressure abnormalities were reported as AEs.

## 4 DISCUSSION

The primary objective of the study was to rule out a higher plasma exposure (in terms of AUC_0–*t*_) of B17MP, formoterol, and GB in adolescents compared to adults, which could therefore indicate a potential safety concern. This was achieved, with the AUC_0–*t*_ values not higher in adolescents than in adults, as indicated by the upper limit of the ratio between adolescents and adults being <125% for all three analytes.

We selected potassium, glucose, and heart rate as the main pharmacodynamic markers as these are known to be rapidly impacted by the administration of inhaled β_2_‐agonist bronchodilators, although predominantly at supratherapeutic doses.^6^ The changes in these values were consistent with the primary endpoint, in that the mean serum potassium concentration was slightly lower, and the mean serum glucose concentration was slightly higher in adults than adolescents. However, the differences between the two groups were small, and few patients had notable changes in potassium, or non‐meal‐related glucose values. The BDP/FF/GB pMDI doses licensed for the maintenance treatment of asthma in adults are 100/6/12.5 and 200/6/12.5 μg, both as two puffs twice daily. This means that the single BDP/FF/GB dose administered in this study (400/24/50 μg) is effectively supratherapeutic for the FF and GB molecules (as the dose administered would usually be divided across two time points), and is the highest approved BDP dose. Furthermore, BDP/FF/GB was overall well‐tolerated, with only three adults (and no adolescents) experiencing AEs—all of which were mild.

Unlike orally‐administered medication, systemic exposure to an inhaled medication does not directly correlate with efficacy, which is mostly driven by topical activity within the lung, and mainly correlates with pulmonary deposition_,_ In the present study, AUC_0–30min_ values (an index of pulmonary deposition) for all three analytes (B17MP, formoterol, and GB) were lower in adolescents than adults. However, the study was not designed to assess therapeutic equivalence, with the early blood sampling in particular not adapted to the rapid pulmonary absorption of FF and GB, and the rapid conversion of the parent compound BDP into B17MP. In addition, given we recruited patients who were receiving maintenance therapy for their asthma, we wished to keep the potential impact of the study on their overall disease control to a minimum by only requiring a 2‐day washout of their maintenance therapy. To properly evaluate relative efficacy would require a suitably designed (and powered) study, with a longer washout period and ideally a multi‐dose treatment period. Additional studies would therefore be needed to draw any conclusion on relative efficacy in the two populations.

The B17MP and formoterol results from this study are broadly consistent with those of a prior study that evaluated the PK of extrafine BDP/FF pMDI in adolescents and adults with asthma.^7^ In this previous study, mean *C*_max_ and AUC_0–30min_ values of both molecules were slightly lower in adolescents than adults, as was AUC_0–*t*_ of B17MP. However, changes in the pharmacodynamic variables (which included peak FEV_1_, so providing a direct assessment of efficacy) were similar in the two age groups. In a second study that evaluated the PK of extrafine BDP/FF pMDI (although delivered using a spacer) in adolescents and adults with asthma (and that also included a group of children aged 7–11 years), systemic exposure to formoterol was again slightly lower in adolescents than adults; exposure to B17MP was slightly higher in adolescents, but all upper 90% CIs of the point estimates of the comparisons between the two age groups remained <125%.^8^ A potential reason for lower systemic exposure in adolescents is that the lungs continue to develop until early adulthood.^9^ The smaller lower airway dimensions in adolescents could potentially result in a lower tidal volume and a shorter time of inhalation, whereas smaller upper airway dimensions could result in a higher proportion of the dose being trapped in the mouth and swallowed.

A key strength of the study is that all patients were trained on the correct use of the pMDI at both the screening visit and pre‐dose on Day 1. The main limitation is that the study was of single‐dose design, and so the parameters were not assessed at steady‐state—although this is mitigated by administration of a supratherapeutic (or high) dose, and multiple dosing was not required to investigate the primary endpoint. Importantly, the good overall safety and tolerability profile of BDP/FF/GB in the two previous 52‐week studies in adults with asthma is reassuring.^2^ In addition, due to the European Medicines Agency recommendation that the volume of blood withdrawn should be minimized in pediatric studies,^10^ blood sampling was limited to 10 h post‐dose, and as a result, the elimination phase of the PK profiles was incompletely characterized.

In conclusion, systemic exposure following administration of a single total dose of extrafine BDP/FF/GB 400/24/50 μg via pMDI in adolescents was not higher than in adults with controlled asthma. Furthermore, there were no relevant differences in terms of the pharmacodynamic parameters, and BDP/FF/GB was well tolerated in both age groups. Overall, therefore, there are no safety signals to warrant a reduction in dose for adolescents with asthma to use BDP/FF/GB. Additional studies, especially multiple‐dose, are needed to evaluate the efficacy of BDP/FF/GB in adolescents with asthma.

## FUNDING INFORMATION

This study was funded by Chiesi Farmaceutici SpA. Writing support was provided by David Young of Young Medical Communications and Consulting Ltd. This support was funded by Chiesi Farmaceutici SpA.

## DISCLOSURE

PK reports personal fees from Chiesi, Novartis, AstraZeneca, Boehringer Ingelheim, Berlin Chemie Menarini, Adamed, Polpharma, and Lekam, all outside the submitted work. JJ declares no conflict of interest. MM and GC were employees of Chiesi Farmaceutici SpA, the sponsor of the study, at the time that the study was conducted, and AV, IB, FM, and GG are employees of Chiesi Farmaceutici SpA.

## ETHICS APPROVAL STATEMENT

The study was approved by the Bioethics Committee at the Medical University of Łódź, Poland, and was performed in accordance with the principles of the Declaration of Helsinki, and the International Conference on Harmonization notes for guidance on Good Clinical Practice (ICH/CPMP/135/95).

## PATIENT CONSENT STATEMENT

All patients provided written informed consent prior to any study‐related procedure.

## PERMISSION TO REPRODUCE MATERIAL FROM OTHER SOURCES

Not applicable.

## Supporting information


Data S1

## Data Availability

Chiesi commits to sharing with qualified scientific and medical researchers, conducting legitimate research, the anonymized patient‐level and study‐level data, the clinical protocol, and the full clinical study report of Chiesi Farmaceutici SpA‐sponsored interventional clinical trials in patients for medicines and indications approved by the European Medicines Agency and/or the US Food and Drug Administration after January 1, 2015, following the approval of any received research proposal and the signature of a Data Sharing Agreement. Chiesi provides access to clinical trial information consistently with the principle of safeguarding commercially confidential information and patient privacy. Other information on Chiesi's data sharing commitment, access, and research request's approval process is available in the Clinical Trial Transparency section of http://www.chiesi.com/en/research‐and‐development/, including the clinical trial data request portal.
