# Supplementary material for: Pharmacokinetics of extrafine beclometasone dipropionate/formoterol fumarate/glycopyrronium bromide in adolescent and adult patients with asthma
Source: Pharmacol Res Perspect. 2022 Jun 22;10(4):e980. doi: 10.1002/prp2.980 (PMC9218519; doi:10.1002/prp2.980)
Supplement: Supplementary file 1 — Data S1 [file PRP2-10-e980-s001.pdf]

Pharmacokinetics of extrafine beclometasone dipropionate/formoterol fumarate/glycopyrronium bromide in adolescent and adult patients with asthma

Piotr Kuna, Joanna Jerzynska, Matteo Martini, Andrea Vele, Irene Barneschi, Fabrizia Mariotti, George Georges, Giorgia Ciurlia

Supplementary methods

Inclusion Criteria

Patients had to meet all of the following inclusion criteria at screening to be eligible for enrolment into the study:

1. Patient's and/or patient legal representative's/parents' (where applicable) written informed consent obtained prior to any study-related procedure.

2. Ability to understand the study procedures, the risks involved, and ability to be trained to use the pMDI device correctly.

3. Male and female adolescents, aged ≥12 and <18 years, or male and female adults, aged ≥18 and <65 years.

4. Body mass index (BMI) within the range of 18.0 to 30.0 kg/m^2, inclusive, and body weight ≥39 kg.

5. A diagnosis of asthma as defined in the GINA guidelines (updated 2019) at least 6 months before the screening visit.

6. Male/female adolescent and adult patients with controlled asthma according to the GINA guidelines (updated 2019) to allow a wash-out period from inhaled BDP of 2 days before study treatment visit.

7. Male/female adolescents and adults with controlled asthma on regular treatment with medium doses of ICS according to the GINA guideline alone or in fixed-dose combinations with LABA and using short-acting inhaled β_2-agonists as a reliever.

8. Adolescents and adults with a forced expiratory volume in one second (FEV_1) >70% of predicted values after withholding short-acting β_2-agonist treatment for a minimum of 6 h prior to screening or 24 h in case of LABA.

9. Non-smokers or ex-smokers who smoked <5 pack-years (pack-years = the number of cigarette packs per day, times the number of years) and stopped smoking >1 year prior to screening.

10. Good physical and mental status, determined on the basis of the medical history and a general clinical examination, at screening and at Visit 1 before dosing.

11. Female patients of non-childbearing potential (WONCBP) defined as physiologically incapable of becoming pregnant (i.e., post-menopausal or permanently sterile) and female patients of childbearing potential (WOCBP) fulfilling one of the following criteria:

    a. WOCBP with fertile male partners: they and/or their partner had to be willing to use a highly effective birth control method from the signature of the informed consent and until the follow-up visit, or

    b. WOCBP with non-fertile male partners (contraception was not required in this case).

    Pregnancy tests were to be performed at study entry in all WOCBP.

    As adolescents of non-childbearing potential could become of childbearing potential during the study, the Investigator had to check the status of the patients during the study and perform pregnancy tests when applicable.

Exclusion Criteria

The presence of any of the following exclusion criteria at screening excluded a patient from study enrolment:

1. Blood donation (equal or more than 450 mL) or blood loss, less than 2 months prior to screening or prior to Visit 1.

2. Abnormal haemoglobin (Hb) level defined as <12.0 g/dL in adolescent male and <12.0 g/dL in adolescent female; <13.0 g/dL in adult male and <12.0 g/dL in adult female.

3. For females only: pregnant and lactating female patients, confirmed by a positive urine pregnancy test at screening or urine pregnancy test at Visit 1 before dosing.

4. Diagnosis of chronic obstructive pulmonary disease (COPD), in adult patients, as defined by the current Global Initiative for Chronic Obstructive Lung Disease (GOLD) guidelines (2019 report).

5. Positive to human immunodeficiency virus type 1 (HIV1) or type 2 (HIV2) or positive results for hepatitis which indicated acute or chronic hepatitis B (i.e., positive hepatitis B surface antigen [HBsAg], positive hepatitis B core antibody [anti-HBc]) or hepatitis C (positive hepatitis C virus antibody).

6. Unsuitable veins for repeated venepuncture.

7. Documented history of alcohol abuse within 12 months prior to screening.

8. Documented history of drug abuse within 12 months prior to screening, or positive urine drug test performed at screening or before dosing.

   Note: In case of an abnormal urine drug test, which could indicate a temporary condition, the test can be repeated once and the results must be available at Visit 1 before dosing. Urine drug test could be repeated once within 4 hours or in a separate visit to avoid a false positive result.

9. Patients who had a positive urine test for cotinine at screening or before dosing.

10. Clinically relevant abnormal laboratory values, which suggested an unknown disease and required further clinical investigation.

11. Clinically relevant and uncontrolled cardiac, hepatic, renal, gastrointestinal, endocrine, metabolic, neurologic, or psychiatric disorder that could interfere with successful completion of this protocol.

12. Known intolerance/hypersensitivity to any of the excipients/components contained in any of the formulations used in the study.

13. Patients with medical diagnosis of narrow-angle glaucoma, prostatic hypertrophy, or bladder neck obstruction that in the opinion of the Investigator prevented use of an anticholinergic.

14. Abnormal 12-lead digitised ECG (12-lead ECG) parameter (i.e., time interval between the beginning of the Q wave and the termination of the S wave [QRS] >120 msec and/or time interval between the onset of the P wave and the beginning of the QRS complex [PR] >210 msec and/or HR <40 bpm and/or HR >110 bpm and/or QTcF >450 msec for males or QTcF >470 msec for females, considering the average from triplicate) or 12-lead ECG evaluated as abnormal clinically significant by the Investigator, at screening.

15. Abnormal blood pressure (BP) (i.e., DBP >90 mmHg and/or SBP >140 mmHg, considering the average from triplicate) at screening.

16. Participation in another clinical study with an investigational drug in the 30 days or 5 half lives of that investigational drug (whichever was longer) preceding the administration of the study drug; a longer and more appropriate time could be considered by the Principal Investigator based on the elimination half-life and/or long-term toxicity of the previous investigational drug.

17. Patients taking enzyme-inducing drugs, enzyme-inhibiting drugs, biologic drugs, or any drug known to have a well-defined potential for hepatotoxicity (e.g., isoniazid, nimesulide, ketoconazole) in the three months before screening or Visit 1.

18. Patients who had a high caffeine intake (>5 caffeinated beverages, e.g., coffee, tea, cola, per day).

19. Patients who had a lower respiratory tract infection (LRTI) within four weeks prior to screening or Visit 1.

20. Patients who were night shift workers with night shifts within eight weeks prior to screening or Visit 1 and during the study.
